# Deficiency of Antinociception and Excessive Grooming Induced by Acute Immobilization Stress in *Per1* Mutant Mice

**DOI:** 10.1371/journal.pone.0016212

**Published:** 2011-01-14

**Authors:** Jing Zhang, Zhouqiao Wu, Linglin Zhou, Huili Li, Huajing Teng, Wei Dai, Yongqing Wang, Zhong Sheng Sun

**Affiliations:** 1 Behavioral Genetics Centre, Institute of Psychology, Chinese Academy of Sciences, Beijing, People's Republic of China; 2 Graduate University of Chinese Academy of Sciences, Beijing, People's Republic of China; 3 Peking University Third Hospital, Peking University Health Science Center, Beijing, People's Republic of China; 4 Institute of Genomic Medicine, Wenzhou Medical College, Wenzhou, Zhejiang, People's Republic of China; 5 Capital Institute of Pediatrics, Beijing, People's Republic of China; Pennsylvania State University, United States of America

## Abstract

Acute stressors induce changes in numerous behavioral parameters through activation of the hypothalamic-pituitary-adrenal (HPA) axis. Several important hormones in paraventricular nucleus of the hypothalamus (PVN) play the roles in these stress-induced reactions. Corticotropin-releasing hormone (CRH), arginine-vasopressin (AVP) and corticosterone are considered as molecular markers for stress-induced grooming behavior. Oxytocin in PVN is an essential modulator for stress-induced antinociception. The clock gene, *Per1*, has been identified as an effecter response to the acute stresses, but its function in neuroendocrine stress systems remains unclear. In the present study we observed the alterations in grooming and nociceptive behaviors induced by acute immobilization stress in *Per1* mutant mice and other genotypes (wild types and *Per2* mutant). The results displayed that stress elicited a more robust effect on grooming behavior in *Per1* mutant mice than in other genotypes. Subsequently, the obvious stress-induced antinociception was observed in the wild-type and *Per2* mutant mice, however, in *Per1* mutant, this antinociceptive effects were partially-reversed (mechanical sensitivity), or over-reversed to hyperalgesia (thermal sensitivity). The real-time qPCR results showed that in PVN, there were stress-induced up-regulations of *Crh*, *Avp* and *c-fos* in all of genotypes; moreover, the expression change of *Crh* in *Per1* mutant mice was much larger than in others. Another hormonal gene, *Oxt*, was up-regulated induced by stress in wild-type and *Per2* mutant but not in *Per1* mutant. In addition, the stress significantly elevated the serum corticosterone levels without genotype-dependent differences, and accordingly the glucocorticoid receptor gene, *Nr3c1*, expressed with a similar pattern in PVN of all strains. Taken together, the present study indicated that in acute stress treated *Per1* mutant mice, there are abnormal hormonal responses in PVN, correlating with the aberrant performance of stress-induced behaviors. Therefore, our findings suggest a novel functional role of *Per1* in neuroendocrine stress system, which further participates in analgesic regulation.

## Introduction

When an individual experiences stressful events, the state of disharmony or threatened homeostasis and various physiological and behavioral changes are triggered. Therefore, stress induces alterations of brain activity and promotes the changes in various function of brain [1]–[3]. Various stressors, including electrical shock, restraint, force swimming and rotation, have been shown to elicit analgesia, which is modulated by oxytocin hormone produced from paraventricular nucleus of the hypothalamus (PVN) [4]–[7]. Acute physical stress exposure induces analgesic effects on many kinds of pain models, such as the hot-plate test [4], tail-flick test [8] and formalin test [9]. Because lots of stressors are noxious and unpredictable, analgesia would appear to be the most appropriate response for adaptation [10]. As an important part of rodent behavioral repertoire, grooming is often related to dearousal following various stressors, and plays a critical role in behavioral adaptation to stress. Generally, in non-stressed situation, grooming is a complex, ethologically rich ritual, which normally performances in a cephalocaudal direction and consists of several stages, but under stress this precise temporal pattern of grooming activity is rewritten, including the variations of frequency in bouts and the total time in durations [11]–[14]. It has been demonstrated that in PVN of mouse, the level of several hormones, such as corticotropin-releasing hormone (*Crh*), arginine-vasopressin (*Avp*) and corticosterone, are important influence factors to the intensity of stress-induced abnormal grooming behavior [12], [15]–[17]. Furthermore, previous studies have shown that physical and psychological stresses are the robust influencers in the abnormality of circadian rhythm [18], [19]. For example, a widely used stress model, immobilization stress produces a phase delay shift of wheel running [20]. Thus, there could be anatomical and functional interactions of neural mechanisms between the systems of stress, circadian rhythm and nociception.

The fundamental molecular mechanism for circadian rhythm is the autoregulatory transcription-feedback loops of the clock genes, such as the positive factors *Bmal1* and *Clock;* and the negative fectors *Pers* and *Crys* [21]. As one of critical negative factor in the transcription-feedback loops of the clock genes, *Per1* has been identified as a molecular marker of entrainment by light, which is a kind of predictable environmental stimuli [22]. Furthermore, the unpredictable stressors, such as acute immobilization and forced swimming, can cause the rapid induction of *Per1* but not *Per2* mRNA in PVN [23] and other peripheral organs of wild type mice [24], suggesting that *Per1* gene expression in these regions could be associated with stress-induced responses. Importantly, the potential stress marker role of *Per1* also implies that there is a novel transcriptional function of *Per1* interacted with several critical neuroendocrine components in stress system [24].

Based on the experimental evidence of relationships between stress, circadian rhythm and nociception, we then asked the core questions in the present study: if *Per1* plays a functional role in stress systems, what are the stress-induced alterations of behavior in grooming and nociception when *Per1* losses its molecular function? In this study, we used the immobilization stress model in different genotypes of mice, including *Per1* mutant, *Per2* mutant and wild-type control, to examine the alterations in grooming and nociceptive behaviors, and further explored the hormonal mechanisms of these behavioral alterations.

## Materials and Methods

These experiments were approved by the Animal Care and Use Committee of the Institute of Psychology of CAS (Beijing, P. R. China; No: A09030). All procedures were conducted in accordance with the “Guide for Care and Use of Laboratory Animals” published by the National Institutes of Health and the ethical guidelines of the International Association for the Study of Pain.

### Experimental animals

The detailed descriptions of the generation and characterization of the *Per1* and *Per2* mutant is described by Zheng et al [25], [26]. Intercrosses between heterozygous (C57 BL/6×129SvEvBrd) F_1_ offspring gave rise to F_2_ homozygous mutants at the expected Mendelian ratio [27]. Mutant and wild-type (M-WT) animals on this mixed back-ground were used in the present study. We also used C57 BL/6 mouse as another wild-type strain, because in several previous studies of circadian rhythm, C57 BL/6 was usually employed. Animals were kept in a 12 hours (hrs) light: 12 hrs dark cycle (light on: 6:00 am; light off: 18:00 pm) for at least 10 days.

### Immobilization stress

As described by the previous study [9], the mice were subjected to immobilization. The restraint was carried out by placing the mouse in a 50 ml corning tube, and adjusting it with an iron nail on the outside, which crossed in the caudal part of the animal. Adequate ventilation was provided by means of holes at the sides of the tubes. The mice were acutely stressed by immobilization for 1 hr. The non-stressed control mice were submitted to the same handling at the same time except for the immobilization procedure. All experiments of stress were started at 14:00 pm. After 1 hr exposure to stress, animals were immediately used to test the different behavioral variations or collect tissues according to the experimental procedures.

### Grooming behavioral observation

After the exposure to stress, animals' grooming behaviors were immediately observed in 1 hr. The general parameters of grooming behavior (the frequency of bouts and the total time of duration) in our observation have been described by previous studies [28]–[30]. The components of grooming behavior includes paw licking, nose or face grooming, head washing, body grooming or scratching, leg licking and tail/genitals grooming [11]–[13]. The behavioral observations of stress-treated (n = 9 for each genotype) and non-stressed mice (n = 9 for each genotype) were performed in the same manner in a double-blinded fashion.

### Mechanical sensitivity test

The response to mechanical stimuli was quantified by measuring the threshold of hindpaw withdrawals to application of von Frey filaments (Touch Test™ Sensory Evaluators, Stoelting Co., USA). Each mouse was placed in a square plexiglass chamber (12 cm ×12 cm ×20 cm) with a metal mesh floor. After 30 minutes (min) acclimation, several levels of force ranging from 0.02 g to 4 g were applied in ascending order to the plantar skin of one hindpaw. Each filament was tested 5 times for approximately 1–2 seconds (sec), with 30 sec intervals between trials. A withdrawal response was considered valid only if the whole hindpaw was removed from the platform after a single stimulation. The threshold was determined by the von-Frey filament which induced withdrawals no less than 5 to 10 stimuli. The baseline mechanical withdrawal threshold was the average of three measurements. The nociceptive behavioral tests of stress-treated (n = 10 for each genotype) and non-stressed mice (n = 10 for each genotype) were performed in the same manner in a double-blinded fashion.

### Thermal sensitivity test

After 30 min acclimation, the 55°C hot plate test was used to measure response latency for heat stimuli. The mice were placed into a plexiglass cylinder of 25 cm diameter on the heated surface. Then, the duration from the start of thermal stimuli on the hindpaw to withdrawal the stimulated hindpaw was recorded as the value of response latency. A cut-off time of 60 sec was set to avoid tissue damage. The nociceptive behavioral tests of stress-treated (n = 10 for each genotype) and non-stressed mice (n = 10 for each genotype) were performed in a double-blinded fashion.

### Tissue collection

Following 1 hr immobilization stress, brains (n = 6 for each genotype) were rapidly removed and placed into a brain matrix (myneurolab, USA) with coronal planes corresponding to the mouse brain atlas of Paxinos and Franklin (2001, second edition). The coronal sections (1.0–1.5 mm), which included hypothalamic areas, were obtained and placed in ice-cold 0.1 M phosphate-buffered saline. The areas of PVN were bilaterally punched out with metal tube of 1 mm diameter, and the pellets were stored at −80°C until mRNA extraction. For non-stressed control mice (n = 6 for each genotype), the same procedure of tissue collection as in stress-treated mice was used.

### RNA isolation and RT real-time qPCR

RNA was isolated and purified from the punched pellets of brain. After isolation and purification of the total RNA, the concentration of each individual total RNA sample was standardized as 250 ng/µl. To generate single-strand cDNA, 2 µg total RNA was used as the starting template for first strand cDNA synthesis, using the PCR cDNA Synthesis Kit (Promega, USA) according to the manufacturer's instructions.

Real-time PCR was performed using the Bio-Rad Laboratories DNA Engine OPTICON 2 system (USA) with SYBR Green detection. PCR primers for each gene were provided in Table 1. Results of the real-time PCR were first normalized through the amount of target gene mRNA in relation to the amount of reference gene mRNA, *Gapdh* gene. For each gene, the values of mRNA expression level in other groups were calibrated to the one in non-stressed M-WT group, which is designated as 1.

**Table 1:** Primer sequences used for real time PCR.

*Gene*	*Direction*	*Primer* (5′ to 3′)	*Annealing temp* (°C)
*Crh*	Fwd.	GGCATCCTGAGAGAAGTCCCTC	60
	Rev.	ACAGAGCCACCAGCAGCATG	
*Oxt*	Fwd.	GCTGTGCTGGACCTGGATATG	60
	Rev.	AGGGCGAAGGCAGGTAGTTC	
*Per1*	Fwd.	TGAGAGCAGCAAGAGTACAAACTCA	60
	Rev.	CTCGCACTCAGGAGGCTGTAG	
*Per2*	Fwd.	GTCCACCTCCCTGCAGACAA	60
	Rev.	TCATTAGCCTTCACCTGCTTCAC	
*Avp*	Fwd.	TCGCCAGGATGCTCAACAC	60
	Rev.	TTGGTCCGAAGCAGCGTC	
*Nr3c1*	Fwd.	CAAGGGTCTGGAGAGGACAA	60
	Rev.	TACAGCTTCCACACGTCAGC	
*c-fos*	Fwd.	CCCCTGTCAACACACAGGAC	60
	Rev.	CCGATGCTCTGCGCTCTGC	
*Gapdh*	Fwd.	TGTGTCCGTCGTGGATCTGA	60
	Rev.	CCTGCTTCACCACCTTCTTGA	

### Determination of corticosterone in serum

Mice (n = 4 for each group) were sacrificed by decapitation following 1 hr immobilization stress, and trunk blood was immediately collected for corticosterone determination. Serum corticosterone levels were determined by Enzyme-linked immunosorbent assay (ELISA) kit (Jiang Lai Biotechnology, Shanghai, China). Sensitivity limit of the assay was 1 ng/ml. The non-stressed control mice were treated by same experimental procedures of blood collecting and corticosterone determination with the stressed mice.

### Data analysis

Data analysis was performed with the software package SPSS 13.0 for Windows (SPSS, Inc., USA). Data were analyzed by one-way and 2×2 two-way (genotype × treatment) ANOVAs. An alpha level of 0.05 was selected for all null hypothesis testing.

## Results

### Grooming behaviors after exposure to immobilization stress in different genotype mice

**Figure 1:**
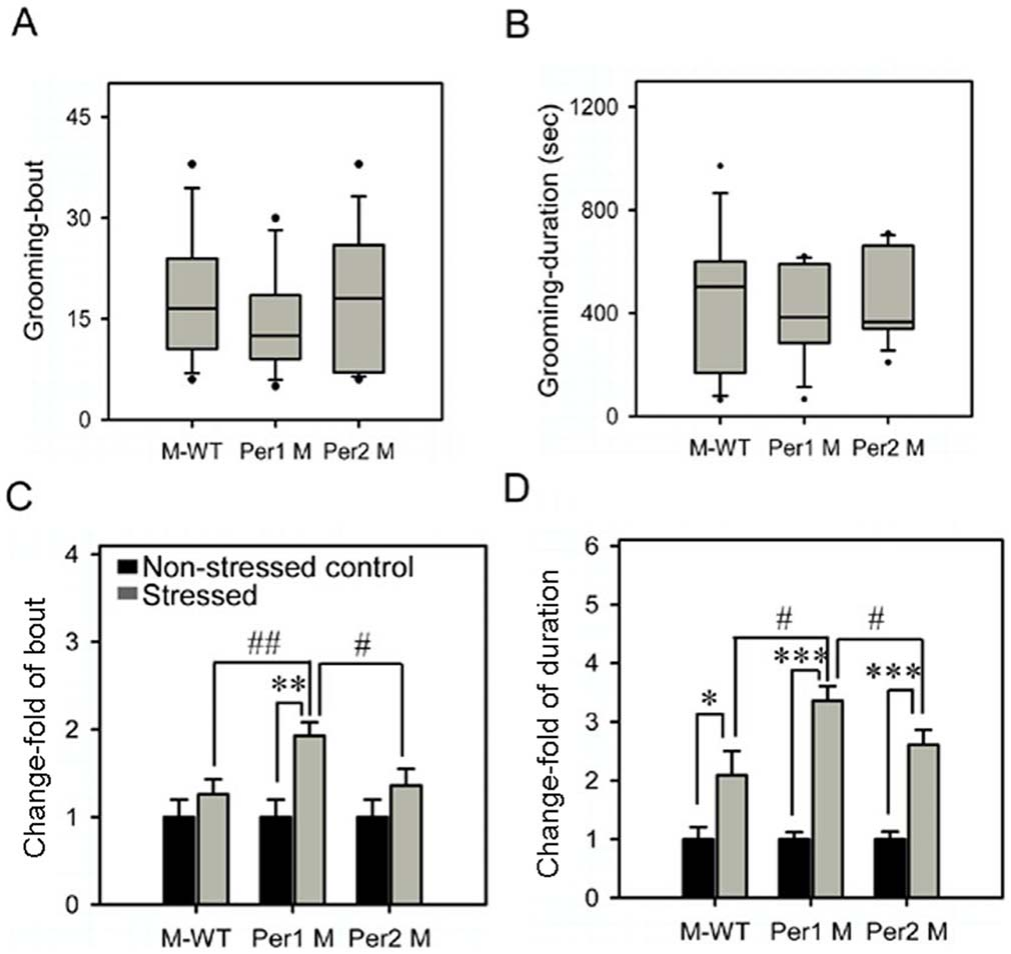
Grooming gross and its change responses to immobilization stress in different genotypes. (A) Number of bouts and (B) Total time of durations of grooming. Box plot graphs represent the distribution of values of the behavioral responses. Boxes extend from the 25th to 75th percentiles and were divided by a solid line representing the median of each group. Whiskers extend from the 5th to 95th percentiles. Each outlier was denoted by a dot. *, *p*<0.05; **, *p*<0.01; ***, *p*<0.001, one way ANOVA. (C) and (D), the changes of grooming bouts and durations in different genotypes. The asterisks (*, *p*<0.05; **, *p*<0.01; ***, *p*<0.001, One way ANOVA) indicate in each genotype, the significant differences in the behavioral responses of the stressed mice compared to the non-stressed mice which is designated as 1. # indicate the significant differences of change-fold in behavioral responses between genotypes (#, *p*<0.05; ##, *p*<0.01; ###, *p*<0.001, one way ANOVA). M-WT, wild-type mice on the mixed back-ground; Per1 M, *Per1* mutant mice; Per2 M, *Per2* mutant mice.

At the basal level, there were no significant difference in both the grooming bouts and grooming durations between the M-WT and other mutant mice (*Per1* mutant and *Per2* mutant) (Figure 1A and 1B), indicating that under non-stressed state, the normal grooming behaviors are kept at the same level in these genotypes.

Following 1 hr immobilization stress exposure, the changes of frequency in grooming bouts and total time in grooming durations were tested in each genotype. The values of stress-treated mice in each genotype were calibrated to the values in non-stressed mice of corresponding genotype, which is designated as 1. The results showed a significantly higher increase fold of the grooming bouts in *Per1* mutant (*F* = 14, *p*<0.01) while the other two genotypes were not affected by stress (Figure 1C). Furthermore, the grooming durations were elevated in all three genotypes after immobilization stress exposure, notably that there was a highest increase fold of grooming durations in *Per1* mutant (Figure 1D). In addition, there was a significant interaction between stress and genetic background (*F* = 19.63, *p*<0.001) in the duration, but in the bouts, this interaction was not significant. These data indicated that the stress induced a more intense grooming behavior in *Per1* mutant mice than in M-WT and *Per2* mutant mice.

### Effects of immobilization stress on nociceptive reactions in different genotypes

**Figure 2:**
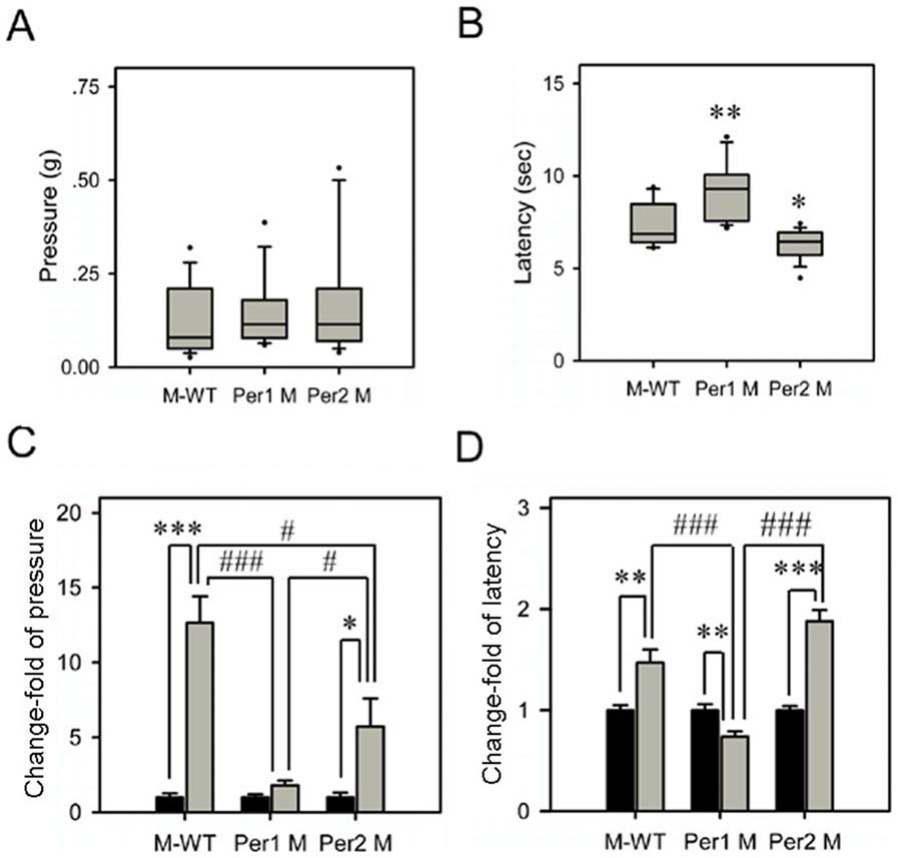
Acute nociception and its change responses to immobilization stress in different genotypes. (A) The withdrawal response to von Frey filament stimuli. (B) The withdrawal latency to thermal stimuli (hot-plate, 55°C). Box plot graphs represent the distribution of values in the behavioral responses. The details referred to legends in Figure 1. In thermal withdrawal latency, the differences in mean response levels between the *Per1* mutant (Per1 M; 9.28 sec), the *Per2* mutant (Per2 M; 6.27 sec) and the mixed back-ground wild-type mice (M-WT; 7.34 sec) were statistically significant: *, *p*<0.05; **, *p*<0.01; ***, *p*<0.001, one way ANOVA. The change-fold of the withdrawal response to von Frey filament stimuli and the withdrawal latency to thermal stimuli are shown in (C) and (D), respectively. The asterisks (*, *p*<0.05; **, *p*<0.01; ***, *p*<0.001, one way ANOVA) indicate in each genotype, the significant differences in the behavioral responses of the stressed mice compared to the non-stressed mice, which is designated as 1. # indicate the significant differences of change-fold in behavioral responses between different genotypes (#, *p*<0.05; ##, *p*<0.01; ###, *p*<0.001, One way ANOVA).

Under the normal state, *Per1* mutant showed similar level of nociceptive response to mechanical stimuli with M-WT and *Per2* mutant (Figure 2A). By contrast, in the hot plate test, *Per1* mutant showed a significant longer latency level than M-WT (*F* = 8.65, *p*<0.01), but *Per2* mutant showed a significant shorter latency level than M-WT (*F* = 5.06, *p*<0.05; Figure 2B).

The changes of nociceptive reactions to mechanical and thermal stimuli after immobilization stress were tested in each genotype (Figure 2C and 2D). Our data showed that in M-WT and *Per2* mutant, the thresholds of response to mechanical stimuli were increased after immobilization stress. In Figure 2C, the change of response to mechanical stimuli in M-WT was 12.65 fold (*F* = 42.7, *p*<0.001), and the one in *Per2* mutant was 5.74 fold (*F* = 6.29, *p*<0.05). However, *Per1* mutant did not show significant change of the response to mechanical stimuli. Furthermore, there was a significant interaction between stress and genetic background (*F* = 17.96, *p*<0.001) in the response to mechanical stimuli.

After stress exposure, the response latency to thermal stimuli were extended in M-WT and *Per2* mutant mice (Figure 2D). The M-WT mice showed a significant change of thermal latency with 1.47 fold (*F* = 10.54, *p*<0.01). Similarly, in *Per2* mutant, the increase fold of thermal latency was 1.88 (*F* = 57.69, *p*<0.001). But notably, contrary to other genotypes, stress-treated *Per1* mutant showed a clear shorter latency to the thermal stimuli than non-stressed (0.73 fold; *F* = 10.8, *p*<0.01, Figure 2D). In addition, there was a significant interaction between stress and genetic background (*F* = 13.4, *p*<0.001) in the response to the thermal stimuli.

These results indicated that immobilization stress produced mechanical and thermal antinociception in both M-WT and *Per2* mutant mice, but these stress-induced antinociceptive effects were attenuated (mechanical nociception), or even reversed to hyperalgesia (thermal nociception) in *Per1* mutant mice.

### Same stress effects on grooming and nociceptive behaviors in M-WT mice and C57 BL/6 mice

**Figure 3:**
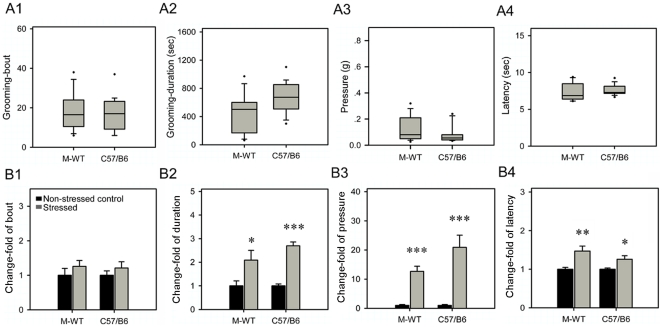
Immobilization stress induced change of grooming and nociceptive behaviors in M-WT and C57/B6 mice. Box plot graphs in A represent the distribution of values of the behavioral responses in non-stressed state. See legend to figure 1 for details. (A1) Number of grooming bouts. (A2) Total time of grooming durations. (A3) Withdrawal response to mechanical stimuli. (A4) Withdrawal latency to thermal stimuli. In basal grooming and nociceptive behavioral values, there were no significant differences between these two strains. Histograms in B represent the changes of grooming gross and nociceptive response after immobilization stress in M-WT and C57/B6 mice. (B1) Grooming bouts. (B2) Duration of grooming. (B3) Mechanical sensitivity. (B4) Thermal sensitivity. The asterisks (*, *p*<0.05; **, *p*<0.01; ***, *p*<0.001, One way ANOVA) indicate in M-WT and C57/B6 mice, the significant differences in the behavioral responses of stressed mice compared to the non-stressed mice, which is designated as 1. In change-fold of all behavioral responses, there were no significant differences between these two strains.

To test the phenotypic differences between M-WT mice and C57 BL/6 mice, we compared their grooming and nociceptive behaviors. As shown in Figure 3A, these two strains exhibited same basal levels of grooming and nociceptive behaviors. In both strains, immobilization stress significantly induced the elevations in grooming duration and the latency of response to mechanical and thermal stimuli, but the increase fold of the behaviors in these two strains were similar (Figure 3B). These data suggested that M-WT mice and C57 BL/6 mice could share similar molecular mechanisms to contribute in the common behavioral phenotypes, and further supported that C57 BL/6 strain could be an appropriate wild type control to *Per* gene mutant strains in previous studies.

### Immobilization stress induced alterations of stress-related genes mRNA expression in PVN

Since our data have shown the genotype-depended divergences of stress-induced behaviors in the mixed back-ground wild type, *Per1* mutant and *Per2* mutant mice, we further investigated the mRNA expression profiles of several stress-related genes (*Crh*, *Avp*, *Oxt*, *c-fos* and *Nr3c1*, which is the encoding gene of glucocorticoid receptor) and *Per* genes in PVN of all strains.

**Figure 4:**
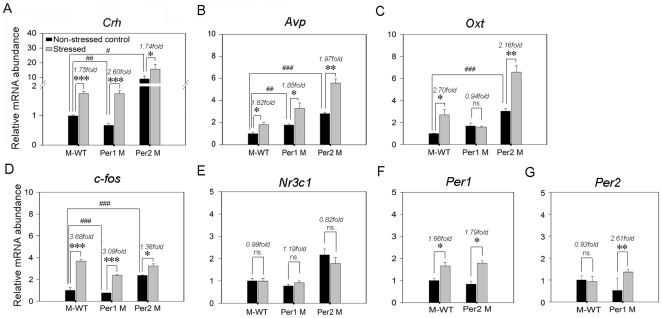
Effects of immobilization stress on the expression of genes in PVN. Relative expression levels and changes (stressed/non-stressed) of stress-related genes (*Crh*, *Avp*, *Oxt*, *c-fos* and *Nr3c1*) and *Per* genes (*Per1* and *Per 2*) in PVN of three genotypes (M-WT, *Per1* mutant and *Per2* mutant) are shown in A–G, respectively. The asterisks (*, *p*<0.05; **, *p*<0.01; ***, *p*<0.001) indicate in each genotype, the significant differences in the gene mRNA expression levels of stressed mice compared to the non-stressed mice, which is designated as 1. # indicate under non-stressed status, the significant differences of relative expression level of genes between *Per* genes mutant mice and M-WT (#, *p*<0.05; ##, *p*<0.01, one way ANOVA).

As Figure 4A shown, under the non-stressed status, the *Crh* mRNA expression level in *Per1* mutant mice was significant lower than the one in M-WT mice (*F* = 20.9, *p*<0.01), by contrast, in *Per2* mutant mice the mRNA expression level was significantly increased (*F* = 14.6, *p*<0.05). After immobilization stress, the *Crh* mRNA expression level were significantly up-regulated in all genotypes (M-WT: 1.75 fold, *F* = 70.7, *p*<0.001; *Per1* mutant: 2.60 fold, *F* = 75.7, *p*<0.001; *Per2* mutant: 1.74 fold, *F* = 9.6, *p*<0.05). Importantly, the increase fold of *Crh* in *Per1* mutant mice was significantly higher than the one in M-WT (*F* = 31.13, *p*<0.01) and *Per2* mutant mice (*F* = 27.67, *p*<0.01).

In Figure 4B, under non-stressed status, the mRNA expression levels of *Avp* were higher in *Per1* mutant (*F* = 22.5, *p*<0.01) and *Per2* mutant mice (*F* = 111.5, *p*<0.001) than the one in M-WT mice. After immobilization stress, the *Avp* mRNA expression level were significantly up-regulated in similar fold in three genotypes (M-WT: 1.82 fold, *F* = 12.04, *p*<0.05; *Per1* mutant: 1.85 fold, *F* = 9.14, *p*<0.05; *Per2* mutant: 1.97 fold, *F* = 45.6, *p*<0.01).

In Figure 4C, under non-stressed state, the mRNA expression level of *Oxt* in *Per1* mutant was similar with the one in M-WT (*F* = 6.3, *p* = 0.07). However, the level in *Per2* mutant was significantly higher than the one in M-WT (*F* = 82.3, *p*<0.001). After immobilization stress, the *Oxt* mRNA expression levels were elevated in both M-WT (2.70 fold, *F* = 12.96, *p*<0.05) and *Per2* mutant (2.16 fold, *F* = 33.35, *p*<0.01). By contrast with the elevations of *Oxt* expression in stressed M-WT and *Per2* mutant mice, there was no significant change in stress treated *Per1* mutant mice (0.94 fold, *F* = 0.12, *p* = 0.75).

In Figure 4D, under non-stressed status, the mRNA expression level of *c-fos* in *Per1* mutant was lower than the one in M-WT (*F* = 72.01, *p*<0.001). However, the level in *Per2* mutant was significantly higher than the one in M-WT (*F* = 713.2, *p*<0.001). After immobilization stress, in all three genotypes the *c-fos* expression levels were siginificantly up-regulated (M-WT: 3.68 fold, *F* = 207, *p*<0.001; *Per1* mutant: 3.09 fold, *F* = 295.8, *p*<0.001; *Per2* mutant: 1.36 fold, *F* = 15.3, *p*<0.05).

The mRNA expression level of *Nr3c1* were not affected by both genotype and stressed-treatment (Figure 4E). Furthermore, under non-stressed state, there was no significant difference of *Per1* gene expression between M-WT and *Per2* mutant mice (*F* = 0.95; *p* = 0.39), but in both genotypes, the gene expression levels were up-regulated after immobilization stress (M-WT: 1.66 fold, *F* = 12.1; *p*<0.05; *Per2* mutant: 1.79 fold, *F* = 15.57; *p*<0.05; Figure 4F). The *Per2* gene expression level was no significant difference between M-WT and *Per1* mutant mice under non-stressed state, however, after immobilization stress the expression level of *Per2* was upregulated in *Per1* mutant mice (2.61 fold, *F* = 40.27, *p*<0.01), but no variation in M-WT (0.93 fold, *F* = 0.05, *p* = 0.83; Figure 4G).

The results of *Crh* expression change response to stress indicated that compared with M-WT and *Per2* mutant mice, there is a more active *Crh* hormonal reaction in PVN of *Per1* mutant mice. However, the results of *Avp* expression suggested that not all hormonal genes were affected by the lack of *Per1*. Importantly, the results of *Oxt* expression indicated that stress probably elicit the activation of oxytocinergic pathway in PVN of M-WT and *Per2* mutant mice, however, this stress-related activation was attenuated in *Per1* mutant mice. Furthermore, for the up-regulation of *Per1* response to stress in M-WT mice, there is coincidence between our data and the results from previous study [23]. We for the first time showed a significant up-regulation of *Per2* gene response to stress in *Per1* mutant, although previous study indicated that this gene is not response to stress in wild type mice [23].

### Effect of immobilization stress on serum corticosterone levels

**Figure 5:**
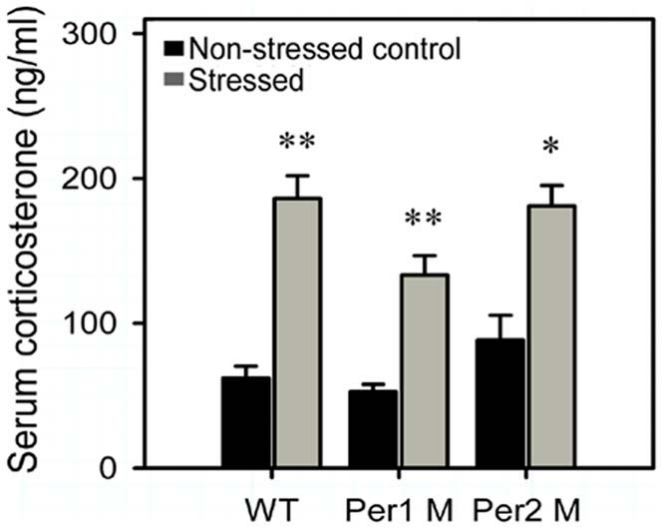
Increased serum corticosterone response to immobilization stress in different genotypes. Serum samples were collected from stressed and non-stressed control of wild type, *Per1* mutant and *Per2* mutant mice, and assayed with the corticosterone ELISA kit. Data shown represent the mean ± SEM of 4 animals from several independent experiments for each data point. The asterisks (*, *p*<0.05; **, *p*<0.01, one way ANOVA) indicate in each genotype, the significant differences of corticosterone level between the stressed mice and the non-stressed mice.

Since corticosterone, as a major stress hormone in rodents, is quickly up-regulated in response to stress in normal wild type mice [1], we then monitored the serum corticosterone levels in our M-WT mice and *Per* genes mutant mice under both non-stressed and stressed statuses. As our results shown (Figure 5), in all non-stressed animals serum corticosterone basal levels had no significant differences between M-WT (62.02±8.2 ng/ml), *Per1* mutant (52.92±4.8 ng/ml) and *Per2* mutant mice (88.34±17.31 ng/ml). Furthermore, after immobilization stress, serum corticosterone levels showed the robust increase in M-WT (186.3±15.5 ng/ml; *F* = 50.29, *p*<0.01), *Per1* mutant (133.25±13.5 ng/ml; *F* = 31.5, *p*<0.01) and *Per2* mutant mice (180.9±14.27 ng/ml; *F* = 17.02, *p*<0.05). Importantly, there were no significant differences in stress-induced increase fold of serum corticosterone levels between these three genotypes (*F* = 2.68, *p* = 0.15).

## Discussion

Previous studies have provided clear evidence for the critical role of PVN hormones in the stress-induced activation of grooming behavior and analgesia [4]–[7], [12], [15]–[17]. In the present study we found that in *Per1* mutant mice the grooming behavior and anti-nociceptive effect induced by immobilization stress showed abnormal alterations compared to wild type mice and *Per2* mutant mice. Moreover, in PVN, for the stress-induced expression changes of hormonal genes, *Crh* and *Oxt*, *Per1* mutant mice also exhibited significant differences from all other genotypes, suggesting that the lack of *Per1* could lead to a functional aberration of hormonal system in PVN, further produce the effects on stress-induced grooming behavior and analgesia.

Many brain regions modulating or mediating activities of neuromediators and hormones are involved in the regulation of grooming behaviors. Recent studies have provided the anatomical and functional evidence that PVN plays a primary role in modulating stress-induced grooming responses [11]–[13]. In PVN neurons, CRH and AVP facilitate stress-induced grooming behavioral activation [12], [15]–[17]. Similarly, our results showed that in M-WT, *Per1* mutant and *Per2* mutant mice, immobilization stress induced significant up-regulations of the stress-related genes *Crh*, *Avp* and *c-fos* in PVN, and the grooming durations were significantly increased, suggesting that there are the general activations of HPA axis in these strains, further supporting the point that *Crh* and *Avp* in PVN are the important modulators to contribute in the arousal mechanisms following stress-induced grooming behavioral activation. However, after stress treatment, there was a significant difference of frequency change in grooming bouts between *Per1* mutant and the two other genotypes, and the increase of *Crh* in *Per1* mutant mice was much stronger than others, implying that *Crh*, rather than *Avp*, might be a critical factor for the difference of frequency change in grooming bouts. The previous study demonstrated that the corticosterone response following stress stimulation is negatively correlated with the amount of grooming behavior [12]. However, our results provided evidence that the stress elevated the serum corticosterone levels without genotype-dependent differences, and the glucocorticoid receptor gene, *Nr3c1*, expressed with a similar pattern in PVN of all strains. We speculate that the negative circulating corticosterone would not contribute in the difference of grooming bouts between *Per1* mutant and the other genotypes.

Stress-induced analgesia is contributed by multiple changes of molecular activity in neural systems, such as endogenous opiate system [31], serotoninergic system [32] and catecholaminergic system [33]. However, recent evidence demonstrated that PVN is an important part of the endogenous pain inhibitory system [5]–[7]. The peripheral stimuli responding by PVN cells specifically are the noxious stimuli, and this type of response defines a specific property of these cells. Furthermore, the results of these PVN cells responding antidromically to spinal cord stimulation and to noxious stimulation suggest that a spinal-hypothalamic-spinal loop, which could be a homeostatic mechanism, participates in pain control [5]. In details, PVN neurons directly project to different central nervous system areas, including the superficial dorsal horn of the ipsilateral spinal cord. Particularly, oxytocinergic descending terminals from the PVN were distributed profusely in the substantia gelatinosa of the superficial dorsal horn [6], [7]. The electrical activation of the PVN or the intrathecal exogenous administration of oxytocin selectively suppresses the incoming A-delta and C fiber afferent nociceptive information, and these effects are reversed by the prior administration of a selective oxytocin antagonist [4], [6], [7]. Moreover, mice lacking oxytocin exhibited significant reduction of stress-induced antinociception following different acute stress [4]. The recent study demonstrated that oxytocin produced from hypothalamic PVN neurons is essential for stress-induced antinociception, and the targets of these inhibitory effects of oxytocin are probably wide dynamic range neurons in spinal dorsal horn [5]. A cellular mechanism for the actions of oxytocin in the spinal cord is that oxytocin inhibits glutamate-mediated sensory synaptic transmission between primary afferent fibers and dorsal horn neurons [6]. Briefly, these results indicated that the PVN oxytocinergic system is involved in pain control through directly binding with its receptor in the spinal cord or activating other neural regulatory mechanisms.

There were several evidence to show the temporal and spatial correlations between the transcriptional activity of *Oxt* mRNA response to stress and the stress-induced specific release of oxytocin by PVN magnocellular neurons [34], [35], suggesting that the stress-induced activation of PVN oxytocinergic system depends on these two aspects. Our behavioral results clearly showed that in the M-WT and *Per2* mutant mice, immobilization stress induced analgesia in both responses to mechanical stimuli and thermal stimuli, but in *Per1* mutant mice, these antinociceptive effects were attenuated (mechanical nociception), even reversed to hyperalgesia (thermal nociception). Furthermore, after immobilization stress *Oxt* was up-regulated in wild-type and *Per2* mutant, but in *Per1* mutant, it remained the non-stressed control level. Therefore, our results suggest that there is an absent of stress-induced oxytocinergic activation in *Per1* mutant mice, and it could be correlated with the deficiency of stress induced antinociception in this strain.

Using the rabbit anti-Per1 antiserum to investigate expression pattern of PER1 in the brain of Syrian hamsters, previous study exhibited that there is a PER1-like immunoreactivity in oxytocinergic cell bodies, axons, and terminals of the hypothalamo-neurohypophyseal system, especially in PVN. This PER1-like immunoreactivity does not show gross changes as a function of time of day or lighting, after pinealectomy, or in association with classical stimuli of oxytocin secretion [36], suggesting that in oxytocinergic cells of PVN, PER1 might play a different role than its own circadian function in suprachiasmatic nucleus. Furthermore, forced swimming, immobilization, and LPS injection caused a rapid induction of *Per1* but not *Per2* mRNA in the PVN CRH neurons, and the increase of *Per1* returned to the basal level 3 hrs after stress application [23]. Immobilization stress also induced a rapid increase in the level of *Per1* mRNA, as a stress-responsive marker, but not in that of *Per2* in mouse peripheral tissues (heart, liver and kidney) [24]. These data suggest that after acute stress, *Per1*, rather than *Per2*, plays a potential role of immediate early gene like *fos* to modulate the functions of *Crh* and *Oxt* in PVN neurons [23].

Experimental evidence shows that a “functional” glucocorticoid-responsive element (GRE) exists in the *Per1* promoter region and this GRE is necessary and sufficient for glucocorticoid signaling to cause a rapid increase in the level of *Per1* mRNA, implying that there should be a third pathway to control of *Per1* transcription. This pathway depends on the glucocorticoid signal in response to stress or other equivalent environmental cues, in addition to the above CREB/CRE pathway by photic stimuli as well as by transactivation via E-box by CLOCK-BMAL1 [24]. Interestingly, in response to stress or other equivalent environmental cues, the glucocorticoid signal induces transcription of *Per1* without correlation with clock regulation [24], suggesting there should be a putative feedback loop between *Per1* gene and the HPA stress systems. Moreover, intracerebroventricular administration of oxytocin significantly attenuates the increase of *Crh* mRNA expression in the PVN in response to immobilization stress [4]. Similarly, our data showed that in *Per1* mutant mice, immobilization stress induced much stronger change of *Crh*, however, the expression of *Oxt* did not show any alteration, suggesting that *Per1* gene would influence the regulatory interaction between oxytocin and CRH.

In summary, in the present study, *Per* genes mutant mice were used to investigate stress-induced behavioral changes of nociception and grooming. For the first time, we have presented evidence showing that *Per1* gene not only is a molecular marker responding to environmental stress stimuli, but also probably as a critical factor to modulate the activity of stress system, further influences various physiological processes. However, the regulating mechanisms on the molecular level between *Per1* and critical hormones in PVN neurons should be focused in the future work.

## References

[1] Ulrich-Lai YM, Herman JP (2009). Neural regulation of endocrine and autonomic stress responses.. Nat Rev Neurosci.

[2] Sandi C (2004). Stress, cognitive impairment and cell adhesion molecules.. Nat Rev Neurosci.

[3] Chrousos GP (2009). Stress and disorders of the stress system.. Nat Rev Endocrinol.

[4] Robinson DA, Wei F, Wang GD, Li P, Kim SJ (2002). Oxytocin mediates stress-induced analgesia in adult mice.. J Physiol.

[5] Condes-Lara M, Martinez-Lorenzana G, Rodriguez-Jimenez J, Rojas-Piloni G (2008). Paraventricular hypothalamic nucleus stimulation modulates nociceptive responses in dorsal horn wide dynamic range neurons.. Neurosci Lett.

[6] Condes-Lara M, Rojas-Piloni G, Martinez-Lorenzana G, Rodriguez-Jimenez J (2009). Paraventricular hypothalamic oxytocinergic cells responding to noxious stimulation and projecting to the spinal dorsal horn represent a homeostatic analgesic mechanism.. Eur J Neurosci.

[7] DeLaTorre S, Rojas-Piloni G, Martinez-Lorenzana G, Rodriguez-Jimenez J, Villanueva L (2009). Paraventricular oxytocinergic hypothalamic prevention or interruption of long-term potentiation in dorsal horn nociceptive neurons: electrophysiological and behavioral evidence.. Pain.

[8] d'Amore A, Chiarotti F, Renzi P (1992). High-intensity nociceptive stimuli minimize behavioral effects induced by restraining stress during the tail-flick test.. J Pharmacol Toxicol Methods.

[9] Seo YJ, Kwon MS, Shim EJ, Park SH, Choi OS (2006). Changes in pain behavior induced by formalin, substance P, glutamate and pro-inflammatory cytokines in immobilization-induced stress mouse model.. Brain Res Bull.

[10] Ford GK, Finn DP (2008). Clinical correlates of stress-induced analgesia: evidence from pharmacological studies.. Pain.

[11] Kalueff AV, Tuohimaa P (2004). Grooming analysis algorithm for neurobehavioural stress research.. Brain Res Brain Res Protoc.

[12] Kruk MR, Westphal KG, Van Erp AM, van Asperen J, Cave BJ (1998). The hypothalamus: cross-roads of endocrine and behavioural regulation in grooming and aggression.. Neurosci Biobehav Rev.

[13] Kalueff AV, Tuohimaa P (2005). The grooming analysis algorithm discriminates between different levels of anxiety in rats: potential utility for neurobehavioural stress research.. J Neurosci Methods.

[14] Eguibar JR, Romero-Carbente JC, Moyaho A (2003). Behavioral differences between selectively bred rats: D1 versus D2 receptors in yawning and grooming.. Pharmacol Biochem Behav.

[15] Lumley LA, Robison CL, Chen WK, Mark B, Meyerhoff JL (2001). Vasopressin into the preoptic area increases grooming behavior in mice.. Physiol Behav.

[16] Buwalda B, Nyakas C, Koolhaas JM, Bohus B (1993). Neuroendocrine and behavioral effects of vasopressin in resting and mild stress conditions.. Physiol Behav.

[17] Caldwell JD, Drago F, Prange AJ, Pedersen CA (1986). A comparison of grooming behavior potencies of neurohypophyseal nonapeptides.. Regul Pept.

[18] Antonijevic I (2008). HPA axis and sleep: identifying subtypes of major depression.. Stress.

[19] Meerlo P, Sgoifo A, Suchecki D (2008). Restricted and disrupted sleep: effects on autonomic function, neuroendocrine stress systems and stress responsivity.. Sleep Med Rev.

[20] Fediuc S, Campbell JE, Riddell MC (2006). Effect of voluntary wheel running on circadian corticosterone release and on HPA axis responsiveness to restraint stress in Sprague-Dawley rats.. J Appl Physiol.

[21] Reppert SM, Weaver DR (2002). Coordination of circadian timing in mammals.. Nature.

[22] Albrecht U, Sun ZS, Eichele G, Lee CC (1997). A differential response of two putative mammalian circadian regulators, mper1 and mper2, to light.. Cell.

[23] Takahashi S, Yokota S, Hara R, Kobayashi T, Akiyama M (2001). Physical and inflammatory stressors elevate circadian clock gene mPer1 mRNA levels in the paraventricular nucleus of the mouse.. Endocrinology.

[24] Yamamoto T, Nakahata Y, Tanaka M, Yoshida M, Soma H (2005). Acute physical stress elevates mouse period1 mRNA expression in mouse peripheral tissues via a glucocorticoid-responsive element.. J Biol Chem.

[25] Zheng B, Larkin DW, Albrecht U, Sun ZS, Sage M, Eichele G, Lee CC, Bradley A (1999). The mPer2 gene encodes a functional component of the mammalian circadian clock.. Nature.

[26] Zheng B, Albrecht U, Kaasik K, Sage M, Lu W, Vaishnav S, Li Q, Sun ZS, Eichele G, Bradley A (2001). Nonredundant roles of the mPer1 and mPer2 genes in the mammalian circadian clock.. Cell.

[27] Albrecht U, Zheng B, Larkin D, Sun ZS, Lee CC (2001). MPer1 and mper2 are essential for normal resetting of the circadian clock.. J Biol Rhythms.

[28] Barros HM, Tannhauser SL, Tannhauser MA, Tannhauser M (1994). The effects of GABAergic drugs on grooming behaviour in the open field.. Pharmacol Toxicol.

[29] Espejo EF (1997). Structure of the mouse behaviour on the elevated plus-maze test of anxiety.. Behav Brain Res.

[30] Moody TW, Merali Z, Crawley JN (1988). The effects of anxiolytics and other agents on rat grooming behaviour.. Ann NY Acad Sci.

[31] LaBuda CJ, Sora I, Uhl GR, Fuchs PN (2000). Stress-induced analgesia in mu-opioid receptor knockout mice reveals normal function of the delta-opioid receptor system.. Brain Res.

[32] Korzeniewska I, Plaznik A (1995). Influence of serotonergic drugs on restraint stress induced analgesia.. Pol J Pharmacol.

[33] Ma S, Morilak DA (2005). Norepinephrine release in medial amygdala facilitates activation of the hypothalamic-pituitary-adrenal axis in response to acute immobilisation stress.. J Neuroendocrinol.

[34] Jezova D, Skultetyova I, Tokarev DI, Bakos P, Vigas M (1995). Vasopressin and oxytocin in stress.. Ann N Y Acad Sci..

[35] Nishioka T, Anselmo-Franci JA, Li P, Callahan MF, Morris M (1998). Stress increases oxytocin release within the hypothalamic paraventricular nucleus.. Brain Res.

[36] Tavakoli-Nezhad M, Tao-Cheng JH, Weaver DR, Schwartz WJ (2007). PER1-like immunoreactivity in oxytocin cells of the hamster hypothalamo-neurohypophyseal system.. J Biol Rhythms.

